# Which AI Sees Like Us? Investigating the Cognitive Plausibility of Language and Vision Models via Eye-Tracking in Human-Robot Interaction

**DOI:** 10.3390/s25154687

**Published:** 2025-07-29

**Authors:** Khashayar Ghamati, Maryam Banitalebi Dehkordi, Abolfazl Zaraki

**Affiliations:** 1 School of Physics, Engineering and Computer Science (SPECS), University of Hertfordshire, Hatfield AL10 9AB, UK; m.banitalebi@herts.ac.uk; 2 Robotics Research Group, University of Hertfordshire, Hatfield AL10 9AB, UK

**Keywords:** human attention modeling, LLMs, VLMs, human-robot interaction, human-computer interaction, personalised large language models, adaptive AI systems, AI agent, short-term memory

## Abstract

As large language models (LLMs) and vision–language models (VLMs) become increasingly used in robotics area, a crucial question arises: to what extent do these models replicate human-like cognitive processes, particularly within socially interactive contexts? Whilst these models demonstrate impressive multimodal reasoning and perception capabilities, their cognitive plausibility remains underexplored. In this study, we address this gap by using human visual attention as a behavioural proxy for cognition in a naturalistic human-robot interaction (HRI) scenario. Eye-tracking data were previously collected from participants engaging in social human-human interactions, providing frame-level gaze fixations as a human attentional ground truth. We then prompted a state-of-the-art VLM (LLaVA) to generate scene descriptions, which were processed by four LLMs (DeepSeek-R1-Distill-Qwen-7B, Qwen1.5-7B-Chat, LLaMA-3.1-8b-instruct, and Gemma-7b-it) to infer saliency points. Critically, we evaluated each model in both stateless and memory-augmented (short-term memory, STM) modes to assess the influence of temporal context on saliency prediction. Our results presented that whilst stateless LLaVA most closely replicates human gaze patterns, STM confers measurable benefits only for DeepSeek, whose lexical anchoring mirrors human rehearsal mechanisms. Other models exhibited degraded performance with memory due to prompt interference or limited contextual integration. This work introduces a novel, empirically grounded framework for assessing cognitive plausibility in generative models and underscores the role of short-term memory in shaping human-like visual attention in robotic systems.

## 1. Introduction

The emergence of LLMs and VLMs and their abilities in tasks involving multimodal perception, semantic understanding, and contextual reasoning affects various domains like robotics. In this case, particularly in socially interactive scenarios, there is growing interest in utilising these models to enhance robots’ cognitive and perceptual functions [1,2]. VLMs like LLaVA can generate rich visual scene interpretations, whilst LLMs provide advanced reasoning abilities which making them attractive candidates for robotic systems that need to perceive, understand, and respond to complex human behaviours. However, a critical question remains: to what extent can these models replicate actual human cognition and behaviour in real-world HRI?

Exploring this question, we focus on one fundamental and measurable aspect of human cognition, visual attention, which plays a central role in perception, social communication, and decision-making. In addition, visual attention guides humans in prioritising stimuli, interpreting interactions, and reacting appropriately to social cues. It is therefore a meaningful proxy for evaluating how closely AI models can mimic human behaviour in HRI [2]. In this study, we use human gaze behaviour, recorded through eye-tracking during a live social HRI scenario, as a benchmark for assessing the alignment between AI-driven attention predictions and real human attention. Our approach consists of two main stages: first, we conduct an empirical study to collect eye-tracking data from participants involved in a multi-party human–human social interaction. This gaze data represents the ground truth for human social attention. Second, we input the video footage of the same interaction into a VLM to extract detailed, frame-by-frame visual descriptions. These descriptions are then passed to several state-of-the-art LLMs, which are prompted to simulate the role of the human participant and estimate where their attention would be directed across the interaction.

Although many of these models are based on similar transformer architectures, they differ substantially in terms of training data, objectives, fine-tuning methods, and multimodal integration strategies. For example, GPT-based models are often trained on broad general-purpose corpora with reinforcement learning from human feedback, whilst other models might be tuned on task-specific datasets or use alternative mechanisms for visual–language alignment. VLMs further vary in how visual and textual modalities are fused, how attention mechanisms are employed across modalities, and what kind of visual grounding is performed. These differences can significantly influence how a model interprets and prioritises elements within a scene. In robotics, socially proper responses are essential, such variability can have deep effects on system performance. Therefore, it is crucial to find which models are suited for mimicking human behaviour, particularly in interactive scenarios where human-like attention and perception are key to successful robot behaviour.

In this work, we aim to find which models produce attention patterns most aligned with human cognition by comparing the AI-generated attention predictions with actual human gaze data. This evaluation not only offers insight into the cognitive plausibility of different AI models but also provides practical guidance for researchers and engineers in selecting appropriate models for use in robotic perception and cognition modules. Ultimately, our work contributes a novel, behaviourally grounded framework for evaluating generative AI models in robotics and highlights the importance of aligning artificial attention mechanisms with human attentional behaviour to enable more natural, effective, and socially intelligent HRI.

## 2. Related Work

### 2.1. Theoretical Foundations of Cognitive Plausibility

Cognitive plausibility refers to the degree to which artificial systems replicate the underlying computational processes, representational structures, and behavioural patterns characteristic of human cognition [3,4]. Unlike mere performance matching, cognitive plausibility demands that AI systems achieve human-like outcomes through mechanisms that align with established principles of human cognitive architecture. This distinction is crucial for developing AI systems that can effectively collaborate with humans, as cognitively plausible systems exhibit predictable, interpretable, and contextually appropriate behaviour.

Visual attention serves as a particularly valuable proxy for cognitive plausibility due to its direct relationship with underlying cognitive processes and measurable behavioural manifestations [5,6]. The eye–mind hypothesis posits that gaze fixations reflect the current focus of cognitive processing, with fixation locations and durations indicating the information being actively processed [7]. The attention–action coupling principle suggests that attentional allocation directly influences subsequent behaviour, making gaze patterns predictive of cognitive intentions [8]. These theoretical foundations provide the basis for using human gaze patterns as benchmarks for evaluating AI cognitive alignment.

The assessment of cognitive plausibility encompasses multiple dimensions of human cognitive processing. Attentional plausibility requires that artificial systems allocate attention according to human-like priorities and mechanisms, reflecting the selective, capacity-limited nature of human attention [9]. Memory plausibility involves replicating the temporal dynamics, capacity constraints, and interference patterns characteristic of human memory systems [10,11]. The integration of these dimensions provides a comprehensive assessment that goes beyond simple performance matching, enabling evaluation of whether AI systems employ computationally similar mechanisms to human cognition.

### 2.2. Computational Models of Visual Attention

Recently, significant efforts have been made to understand and model human visual attention, particularly within interactive social scenarios involving robots. A foundational study by [12] introduced a computational gaze-control system driven by empirical human gaze data collected via eye-tracking to present an insight into aligning robotic perception systems with human visual attention, highlighting the importance of understanding gaze behaviours in HIRs. Additionally, the framework proposed in [13], which has deeply influenced our understanding of social attention mechanisms, emphasises their role in effective social communication and cognitive behaviours. Building upon these theoretical foundations, [14] explored advanced computational models that incorporate semantic and contextual information. These studies collectively contributed to a broader finding of the cognitive significance of attention mechanisms. However, they primarily focused on singular computational models or isolated theoretical analyses, lacking comprehensive empirical validations within dynamic, multimodal interaction contexts.

### 2.3. Advances in Large Language Models and Vision–Language Integration

Concurrently, advances in LLMs have significantly enhanced our capabilities to simulate human cognitive behaviours and complex reasoning processes. The authors of [15] present this by evaluating the ability of models like GPT-4 to encode and utilise expert clinical knowledge, suggesting imperceptible alignments between artificial and human cognitive processes. Similarly, ref. [16] proposed a landmark by demonstrating GPT-3’s remarkable few-shot learning capabilities, effectively simulating diverse human cognitive behaviours. Extending these insights, ref. [17] explored emergent reasoning and cognitive abilities in LLMs, particularly through novel prompting methods such as chain-of-thought, emphasising the models’ potential for deeper cognitive alignment.

Additionally, the vision and language models integration has been increasingly recognised as crucial for accurately modelling complex multimodal human cognition. LLaVA was introduced as a fine-tuned VLM to enhance visual reasoning capabilities, thereby aligning visual perception with linguistic understanding more effectively [18]. In addition, ref. [19] integrated visual foundation models with ChatGPT-3 to facilitate interactive multimodal dialogues for mimicking natural human communication processes. Moreover, ref. [20] discussed the abilities of cognitive grounding using LLMs through simulations, highlighting the potential for language models to replicate human perceptual reasoning.

### 2.4. Research Gaps and Contributions

Although there are these significant advances, notable research gaps remain. The existing literature has largely examined cognitive alignment either in abstract theoretical constructs or within isolated computational frameworks, rarely extending evaluations into real-world interactive scenarios. Comprehensive empirical validations integrating multimodal information, specifically visual and linguistic data, within dynamic HRI contexts are especially limited. Additionally, temporal context and memory dynamics, which are fundamental to human cognitive processes, have been largely overlooked or insufficiently explored in existing evaluations.

This work introduces a novel evaluation framework that comprehensively assesses the cognitive plausibility of various LLMs and a VLM using human gaze data captured via eye-tracking to address these gaps. Our work extends beyond static or isolated scenarios by embedding evaluations within realistic HRI settings. By incorporating STM analysis, we explicitly investigate the impact of temporal context on models’ cognitive alignment, offering deeper insights into the underlying processes of visual attention and human cognition.

Thus, our study successfully fills existing gaps by not only advancing theoretical and computational understandings but also rigorously evaluating cognitive plausibility within dynamic, real-world multimodal interaction scenarios. Our comprehensive empirical results will significantly contribute to developing socially intelligent robotic systems capable of authentically replicating human-like visual and cognitive behaviours in real-time interactive contexts.

## 3. Human Social Attention Exploration: Experimental Study

The development of our proposed model is grounded in empirical insights derived from prior investigations into human attention patterns during social interactions, as detailed in [12]. In particular, we employed eye-tracking methodologies to systematically record and analyse the gaze dynamics of participants observing video sequences of dyadic conversations. This approach facilitated a fine-grained understanding of how individuals distribute their attention across diverse social cues, including both verbal communication and non-verbal gestures.

Participants: This study involved 11 participants recruited from the Department of Mechanical Engineering at the Technical University of Munich—nine male and two female—with a mean age of 27.3 years (range: 22–35 years). Participation was incentivised through the provision of chocolate. All participants provided informed consent for data collection and subsequent analysis, including the current research application.

Experimental Protocol: During the experiment, participants viewed a video depicting two individuals engaged in a discussion on various research topics. The scenario was structured to include naturalistic elements: sequential room entry, seated discussion, and individual departures. To heighten engagement, the interlocutors occasionally addressed the camera directly, simulating a triadic interaction and suggesting the presence of a third party. The complete session lasted 7 min and 20 s and was recorded using an eye-tracker. It was employed comprising a scene camera to capture the visual field and an infrared camera to monitor participants’ left-eye movements. Participants were seated approximately 75 cm from a 23-inch monitor in a controlled laboratory environment. Prior to each session, the system was carefully calibrated for each participant to ensure accurate pupil tracking. All sessions were conducted under standardised lighting conditions to minimise interference from ambient light sources.

Data Processing: Gaze recordings were annotated frame-by-frame in ELAN to mark fixations on Person A (left), Person B (right), or Environment regions, and to note when the non-speaking individual produced a non-verbal cue. Log files of fixation durations (ms) and targets were exported for analysis. We computed the average attention proportions to each person during speaking and non-verbal-cue intervals as well as over the entire video. The aggregated gaze trace revealed both high-frequency (saccadic) and low-frequency (non-saccadic) shifts. For instance, 100% of participants immediately looked at Person A upon entry, and 82% shifted to Person B upon that person’s arrival.

### Study Scope and Limitations

The current investigation represents a foundational study establishing a novel evaluation framework for assessing cognitive plausibility in AI systems through human gaze benchmarks. The empirical evaluation is deliberately constrained to a controlled dyadic interaction scenario to enable rigorous methodological validation whilst maintaining experimental control. This controlled approach, whilst limiting the immediate generalisability of findings, provides several methodological advantages essential for framework establishment.

The single-video scenario involving two individuals engaged in seated conversation represents a well-defined interaction context that facilitates systematic comparison across AI models whilst minimising confounding variables. The 11-participant sample size, whilst modest, aligns with established practices in eye-tracking research where within-subject designs and controlled stimuli enable robust statistical analysis [5,6].

The constrained experimental scope reflects a deliberate methodological choice to establish proof-of-concept for the evaluation framework rather than immediate broad generalisation. This approach parallels established practices in cognitive psychology and HRI research, where controlled laboratory studies provide foundational insights that inform subsequent field validation [21,22,23]. The controlled setting enables precise measurement of gaze patterns, systematic manipulation of memory conditions, and rigorous statistical analysis that would be challenging to achieve in more naturalistic environments.

However, we acknowledge that this controlled approach limits the immediate applicability of our specific findings to diverse HRI contexts. The single-interaction scenario cannot capture the full complexity of real-world human–robot interactions, including multi-agent scenarios, task-oriented behaviours, dynamic environmental conditions, or cultural variations in social attention patterns. Future validation studies employing diverse interaction contexts, larger participant samples, and varied demographic populations will be essential for establishing the broader applicability of our framework.

## 4. Proof-of-Concept Study

**Figure 1:**
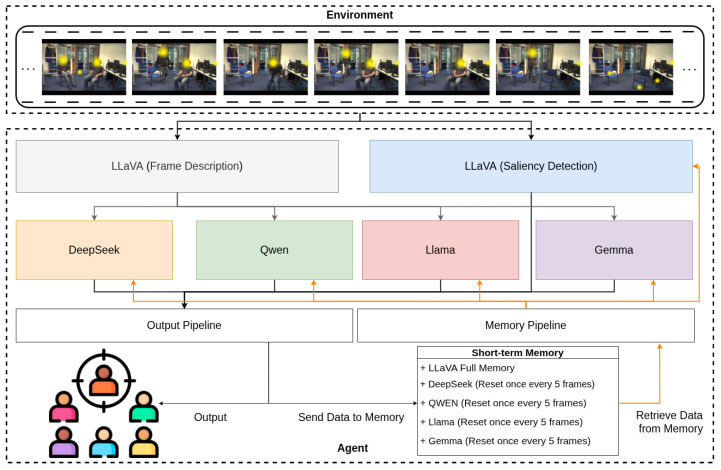
Overview of the evaluation pipeline. The vision–language model processes each video frame to generate textual descriptions, which are passed to four large language models. The system operates in two modes: stateless (frame-by-frame) and memory-augmented (STM), where concatenated frame descriptions are provided. Cosine similarity with human-annotated gaze data is used to assess alignment between model predictions and human saliency. The highlighted points in the frames show the attention pattern of human participant observers. The implementation source code can be find at https://ghamati.com/seelikeus/ (accessed on 15 June 2025).

This study presents a comparative analysis of one VLM, LLaVA [18], and four LLMs, DeepSeek [24], Qwen [25], Llama [26], and Gemma [27], to determine their suitability for robotic applications. The use case centres on identifying saliency points within an environment—a fundamental capability for interactive and perceptually aware robotic systems. This experiment identified areas of interest (saliency points) selected by human observers and subsequently proposed a computational model simulating the human visual attention system. We replicate the same scenario using the corresponding video footage, but rather than relying on human participants and eye-tracking, we employ a VLM and four LLMs to analyse each video frame and determine salient regions. Figure 1 illustrates our agentic system, which runs all models in parallel for performance comparison.

We investigate two analytical approaches. The first involves processing each frame independently to predict the saliency point. The second incorporates a short-term memory mechanism by aggregating outputs across five consecutive frames. Specifically, LLaVA is employed to generate descriptive captions for each frame sequentially. Descriptions from five consecutive frames are concatenated and input into each of the four LLMs to identify salient regions, thereby allowing the models to reason over short-term temporal context.

Additionally, we evaluate LLaVA’s own saliency detection capabilities under two settings: (1) processing frames individually, and (2) sequentially incorporating previously detected saliency outputs into the prompt for subsequent frames. In the latter method, the output from frame *t* is retained and passed along with frame t+1 to LLaVA, with a prompt modified to inform the model of prior context. This iterative process continues until the final frame, effectively enabling LLaVA to track changes in saliency over time.

All results are evaluated against the average human gaze model obtained in [12] to estimate each model’s alignment with human visual attention.

### 4.1. Vision–Language Model

We utilise LLaVA for two primary purposes. First, to generate textual descriptions of each incoming frame, which are subsequently passed to the LLMs for saliency detection (image-to-text pipeline); and second to directly identify the saliency point from each frame using an alternative prompt designed specifically for saliency detection.

For the first use case, each frame is fed into LLaVA with a general instruction prompt to describe the visual content. The resulting textual descriptions are then provided as input to the four LLMs for further saliency analysis. In the second use case, each frame is input directly into LLaVA using a distinct saliency-focused prompt to extract the salient regions.

Table 1 outlines the two types of prompt styles employed in this process. Moreover, to enable LLaVA to incorporate temporal context, we re-invoke the model after the initial saliency detection. The outputs from previous frames are passed as a form of STM to inform the processing of the subsequent frame, allowing for more consistent and temporally aware saliency predictions. This mechanism is visualised in Figure 1, where the orange arrow indicates the flow of contextual information from prior frames to the model.

Specifically, after each frame is processed for saliency, the output is sent both to the visual interface for user inspection and to the STM module for storage. In the memory-augmented saliency detection mode, LLaVA receives the cumulative saliency outputs from frames $t_{0}$ to *t*, along with frame t+1, to assess whether incorporating prior context improves the precision of saliency identification compared to single-frame processing. Technical Constraints and Memory Configuration: The different memory implementations between LLaVA (full cumulative memory) and LLMs (STM-5) were necessitated by computational limitations. During preliminary testing, DeepSeek and Qwen reached GPU memory saturation after 5 cumulative frames, LLaMA after 9 frames, and Gemma after 7 frames, while LLaVA demonstrated sufficient memory efficiency for full 60-frame evaluation. The STM-5 configuration represents the maximum context length universally supported across all LLMs, whilst LLaVA’s superior computational efficiency enabled evaluation under both bounded and extended memory conditions. The following subsections detail the implementation of the agent and the methodologies employed to derive the results.

### 4.2. Large Language Models and Cognitive Memory Architecture

We employed four LLMs: DeepSeek, Qwen, LLaMA, and Gemma, each representing a lightweight version capped at a maximum of 8 billion parameters to ensure the feasibility of self-hosted deployment. Table 2 summarises the models and their configurations. Each LLM was utilised in two experimental configurations designed to assess temporal context integration capabilities and cognitive alignment with human memory processing patterns.

Cognitively-Informed Temporal Context Framework: Our temporal context implementation incorporates several principles derived from cognitive memory research to approximate human short-term memory processing. The sliding context window maintains a bounded memory buffer of five recent frames, reflecting capacity limitations observed in human working memory [11]. This bounded approach prevents unlimited context accumulation that would violate cognitive plausibility principles, whilst enabling systematic assessment of how temporal context affects saliency detection across different architectural paradigms.

The five-frame window provides temporal coherence whilst respecting both computational constraints and cognitive plausibility principles. This design choice aligns with established findings that human visual attention benefits from recent contextual information whilst showing interference effects when memory load exceeds capacity limits [28]. The temporal aggregation enables models to leverage recurring visual patterns and maintain attentional consistency across brief temporal intervals, mimicking the human tendency to anchor attention on stable visual elements whilst adapting to dynamic scene changes.

Architectural Diversity and Cognitive Strategies: The selected models represent diverse approaches to language processing and temporal integration, enabling systematic evaluation of how different architectural strategies handle bounded memory constraints. DeepSeek’s distilled architecture provides efficient context processing optimised for rapid inference, whilst Qwen’s multilingual training enables assessment of cross-lingual cognitive alignment. LLaMA’s instruction-tuned architecture offers insights into how fine-tuning affects temporal reasoning capabilities, whilst Gemma’s compute-efficient design reveals how resource constraints influence memory integration performance.

In each iteration, LLaVA-generated frame descriptions were passed to all four LLMs in parallel, accompanied by either stateless prompts for immediate attention assessment or temporally augmented prompts incorporating the sliding context window for memory-enhanced processing. This parallel evaluation design enables direct comparison of how different architectural approaches handle temporal context integration, providing insights into which models demonstrate human-like benefits from bounded memory versus those that suffer from context interference effects.

The sliding context window implementation treats recent frames as contextual priors that inform current attention allocation, similar to how human visual attention leverages recent gaze history to maintain stable focus on relevant targets whilst adapting to changing visual dynamics. This approach enables systematic evaluation of temporal context effects whilst maintaining cognitive plausibility through bounded memory constraints that parallel human working memory limitations. The temporal context provides models with access to recent attention history, enabling detection of recurring visual elements and maintenance of attentional coherence across temporal sequences.

Memory Integration Assessment: The experimental design specifically examines how each model integrates temporal context with immediate visual information, assessing whether the integration demonstrates cognitively plausible patterns such as improved consistency for stable visual elements, appropriate forgetting of irrelevant information, and adaptive attention allocation based on changing visual dynamics. This evaluation framework provides insights into which architectural approaches most closely approximate human cognitive strategies for temporal attention integration in dynamic social environments.

Table 3 presents the prompts employed for both the sliding context window and stateless configurations, designed to enable fair comparison whilst accommodating the specific capabilities and constraints of each model architecture. The prompt design maintains consistent evaluation criteria whilst allowing each model to leverage its architectural strengths for optimal performance in both immediate attention and temporal integration tasks.

### 4.3. Memory Implementation Design, Technical Constraints, and Computational Infrastructure

The asymmetric memory implementations between LLaVA (cumulative full memory) and LLMs (sliding context window) were necessitated by significant differences in computational efficiency and memory requirements across models. During preliminary testing, we observed that LLaVA could process cumulative memory contexts without GPU memory saturation throughout the entire 60-frame sequence. However, the LLMs exhibited severe memory limitations when processing extended contexts: DeepSeek and Qwen reached GPU memory saturation after 5 frames of cumulative context, LLaMA after 9 frames, and Gemma after 7 frames.

These hardware constraints prevented fair comparison using identical memory implementations across all models. The sliding context window of five frames was selected as the maximum feasible context length that all LLMs could process reliably without memory overflow. Conversely, LLaVA’s superior memory efficiency allowed testing of both bounded and unbounded temporal contexts to assess the impact of extended context on vision–language models. This computational asymmetry reflects fundamental architectural differences: LLaVA’s integrated vision–language processing appears more memory-efficient for temporal sequences, whilst pure language models require significantly more GPU memory when processing concatenated textual contexts of equivalent temporal span.

Our experimental framework operates within a carefully controlled computational environment designed to ensure reproducibility whilst maintaining consistency across all model evaluations. The computational infrastructure employs dual NVIDIA RTX A6000 GPUs with 48 GB VRAM each (total 96 GB), providing substantial memory capacity for simultaneous deployment of multiple language models whilst maintaining stable inference performance. The system operates with CUDA 12.2 and driver version 535.230.02.

Environment validation procedures enable systematic verification of implementation consistency across computational environments. Baseline validation processes predetermined frames with documented expected outputs to verify model configuration accuracy and prompt formatting consistency. Automated consistency checks ensure TF-IDF vectorisation produces identical results across different computational environments, whilst reference similarity calculations provide implementation verification benchmarks for systematic replication validation.

Beyond computational constraints, our memory implementations exhibit fundamental limitations in cognitive plausibility. The sliding context window approach for LLMs and cumulative memory for LLaVA both lack essential characteristics of human memory systems. Human short-term memory operates through capacity-limited, selective, and temporally sensitive mechanisms that our implementations do not capture [28,29]. Our concatenation-based approach treats all temporal information equally, exhibits no forgetting mechanisms, and provides no selective attention processes for filtering relevant from irrelevant information. These limitations mean that observed "memory effects” may reflect simple responses to increased textual context rather than genuine cognitive alignment with human memory processes.

We acknowledge that this asymmetric design limits direct comparability between LLaVA and LLM memory configurations, whilst the simplified memory implementations constrain claims about cognitive plausibility in temporal processing. The hybrid deployment approach, combining local GPU inference with external API services, introduces additional variables but enables comprehensive architectural comparison across diverse computational paradigms. Future work should investigate more sophisticated memory mechanisms that better approximate human cognitive architecture whilst addressing computational constraints through alternative approaches. The comprehensive technical specification provided enables systematic replication whilst offering flexibility for adaptation to alternative computational resources, ensuring that observed cognitive alignment patterns reflect genuine architectural differences rather than implementation variations.

### 4.4. Multimodal Attention Evaluation and Cognitive Alignment Assessment

Our comprehensive evaluation framework integrates multiple assessment approaches to provide robust measurement of cognitive alignment between AI models and human attention patterns. The methodology combines semantic similarity assessment, statistical validation, and temporal coherence analysis to evaluate both immediate attention accuracy and memory integration capabilities across different AI architectures.

Semantic Attention Alignment Framework: We employ TF-IDF cosine similarity as a principled approach for assessing semantic alignment between AI-generated attention descriptions and human gaze patterns. This text-based similarity metric captures the conceptual overlap between AI attention allocation and human visual focus, enabling comparison across different linguistic expressions of attention whilst maintaining sensitivity to semantic content and attentional relevance. The TF-IDF vectorisation approach transforms both AI outputs and ground truth labels into high-dimensional semantic representations that capture the importance and distinctiveness of attention-related terms.

Formally, for each frame *i*, the similarity score is computed as:

(1) $${\mathrm{Score}}_{i}=\cos\left(\theta \right)=\frac{{\vec{v}}_{\mathrm{model},i}\cdot {\vec{v}}_{\mathrm{label},i}}{∥{\vec{v}}_{\mathrm{model},i}∥\;∥{\vec{v}}_{\mathrm{label},i}∥}$$

where ${\vec{v}}_{\mathrm{model},i}$ and ${\vec{v}}_{\mathrm{label},i}$ are the TF-IDF vectors of the model prediction and the ground-truth label. This approach enables robust comparison between varied linguistic expressions of attention (e.g., “man in dark clothing” vs. “person wearing black shirt”) whilst penalising irrelevant or distracting content. The cosine similarity metric provides scale-independent comparison that treats both brief and verbose outputs equitably, focusing on semantic content rather than output length or stylistic variations.

Cross-Modal Cognitive Assessment and Ground Truth Integration: The evaluation framework addresses the fundamental challenge of comparing discrete AI attention descriptions with categorical human gaze data through systematic semantic grounding and cognitive alignment assessment. Human gaze categories (Person A, Person B, and Environment) are expanded into rich textual descriptions based on frame-specific visual characteristics, enabling meaningful comparison with AI-generated attention descriptions whilst preserving the semantic intent of human attention allocation.

The categorical-to-textual mapping process involves systematic annotation of each frame’s salient target based on visual content analysis, with descriptive labels generated according to visible characteristics and spatial context. AI descriptions mentioning specific individuals are mapped to corresponding person categories based on visual verification of clothing, position, and actions visible in each frame. Environment-related descriptions are systematically mapped to the Environment category based on object identification and spatial reference. This mapping process was conducted independently by two researchers to ensure consistency and reduce subjective interpretation bias.

Temporal Context Evaluation and Memory Assessment: To evaluate the contribution of temporal context and assess memory-like processing capabilities, each model was tested under multiple configurations designed to reveal cognitive alignment patterns. The stateless configuration processes each frame independently, providing baseline attention accuracy without temporal context. The STM-5 configuration provides models with concatenated descriptions of the preceding four frames alongside the current frame, enabling assessment of bounded memory integration capabilities. The full-memory configuration, applied exclusively to LLaVA, accumulates complete temporal context throughout the sequence to assess the effects of unlimited context accumulation.

We utilise the following analytical framework to quantify temporal context effects:

(2) $$\Delta_{i}\left(m\right)=s_{i}\left(m,\mathrm{memory}\right)-s_{i}\left(m,\mathrm{stateless}\right)$$

(3) $$s_{i}\left(m\right)=\frac{1}{3}\left(s_{i-1}\left(m\right)+s_{i}\left(m\right)+s_{i+1}\left(m\right)\right)$$

where $\Delta_{i}\left(m\right)$ represents the instantaneous cognitive benefit or cost attributable to memory integration for model *m* and frame *i*, whilst $s_{i}\left(m\right)$ provides temporal smoothing to suppress transient effects and reveal underlying cognitive patterns. This framework enables systematic assessment of whether models demonstrate human-like benefits from bounded memory or exhibit non-cognitive patterns such as unlimited context accumulation without capacity constraints.

Advanced Statistical Validation Framework: Our statistical analysis employs sophisticated non-parametric approaches designed specifically for the bounded, non-normal distribution of similarity scores observed in attention evaluation studies. Initial examination revealed significant departures from normality and heteroscedasticity across model conditions, necessitating robust statistical approaches that account for these distributional characteristics whilst providing valid inference about cognitive alignment patterns.

We employed a repeated-measures design treating the 60 video frames as the unit of analysis, with each frame evaluated across ten model–regime conditions including LLaVA-stateless, LLaVA-full memory, DeepSeek-stateless, DeepSeek-STM, Qwen-stateless, Qwen-STM, LLaMA-stateless, LLaMA-STM, Gemma-stateless, and Gemma-STM. This approach accounts for the inherent dependency structure where identical visual content is evaluated by all models, providing greater statistical power than independent-samples designs whilst controlling for frame-specific variance that might otherwise confound cognitive alignment assessment.

The analysis commenced with a Friedman test to evaluate the global null hypothesis that all model–regime conditions demonstrate equivalent cognitive alignment. This non-parametric equivalent of repeated-measures ANOVA assesses whether median similarity scores differ significantly across conditions whilst accounting for temporal dependencies and individual frame characteristics. The omnibus Friedman test yielded highly significant results, $\chi^{2}\left(9\right)=215.8,\;p<0.001$, indicating substantial cognitive alignment differences warranting detailed pairwise examination.

Following significant omnibus results, we conducted targeted pairwise comparisons using Wilcoxon signed-rank tests for theoretically motivated contrasts that address specific cognitive alignment hypotheses. These included comparisons between immediate attention accuracy (stateless conditions), memory integration capabilities (within-model memory effects), and cross-model cognitive strategy assessment. All pairwise comparisons employed Holm–Bonferroni adjustment to control family-wise error rates whilst maintaining statistical power for detecting meaningful cognitive alignment differences.

Effect sizes were calculated using $r=Z/\sqrt{N}$ where N=60 frame pairs, providing standardised measures of practical significance that quantify the magnitude of cognitive alignment differences beyond statistical significance. Following Cohen’s conventions, r=0.10 indicates small cognitive alignment differences, r=0.30 indicates medium differences, and r=0.50 indicates large cognitive alignment differences with substantial practical implications for human-robot interaction applications.

Cognitive Plausibility Metrics and Human Alignment Assessment: The evaluation framework incorporates multiple cognitive plausibility indicators that assess whether AI models demonstrate human-like attention strategies rather than arbitrary performance patterns. These include temporal consistency measures that assess whether models maintain stable attention allocation for persistent visual elements, adaptive attention measures that evaluate appropriate attention shifting for dynamic visual content, and memory integration measures that assess whether temporal context provides cognitively plausible benefits without violating capacity constraints.

Key Cognitive Alignment Findings: The statistical analysis reveals several critical insights about cognitive architecture differences, as detailed in Table 4. LLaVA’s immediate attention superiority over the best memory-enhanced competitor (DeepSeek-STM) achieves a large effect size (r=0.63), establishing a clear cognitive processing hierarchy amongst the evaluated architectures. The comparison between LLaVA’s stateless and full-memory conditions demonstrates a substantial negative impact (r=0.70) from unlimited context accumulation, providing strong statistical evidence for cognitively plausible capacity limitations. DeepSeek’s reliable improvement from bounded memory integration (r=0.55) confirms that appropriate temporal context can enhance language-centric architectures without causing interference effects.

The framework enables systematic assessment of cognitive strategies employed by different AI architectures, providing insights into which approaches most closely approximate human cognitive mechanisms for attention allocation and temporal processing in dynamic social environments. This cognitive assessment extends beyond simple performance measurement to evaluate the underlying strategies and mechanisms that drive attention allocation, enabling identification of models that achieve performance through cognitively plausible rather than arbitrary computational approaches.

### 4.5. AI Output to Human Gaze Mapping and Evaluation Methodology

Our evaluation framework compared AI-generated textual saliency predictions against categorical human gaze data from the original eye-tracking study. The LLMs and VLMs generated natural language descriptions of salient regions such as “man in black shirt,” “wooden table,” or “person gesturing,” whilst the human ground truth consisted of categorical fixation targets (Person A, Person B, and Environment) derived from frame-by-frame gaze analysis. This comparison required systematic mapping between AI textual outputs and categorical human gaze targets, representing a fundamental methodological challenge in multimodal evaluation studies.

To enable quantitative comparison, we developed a systematic mapping protocol between AI textual outputs and categorical human gaze targets. AI descriptions mentioning specific individuals were mapped to the corresponding person category based on visual verification of clothing, position, and actions visible in each frame. For instance, AI-generated descriptions such as “man in black shirt” were mapped to Person B when that individual was indeed wearing dark clothing in the corresponding frame. Similarly, environment-related descriptions including “table,” “monitor,” or “room” were systematically mapped to the Environment category. This mapping process was conducted independently by two researchers to ensure consistency and reduce subjective interpretation bias.

Following the mapping process, we converted both AI outputs and the mapped categorical labels into TF-IDF vectors for cosine similarity calculation. The categorical labels were expanded into standardised textual descriptions based on frame-specific visual characteristics to enable meaningful lexical comparison. For example, “Person A” was expanded to “man in striped shirt” when that individual’s clothing represented the most visually prominent feature in the corresponding frame. This expansion process maintained consistency with the original categorical annotations whilst providing the textual representation necessary for TF-IDF analysis.

Cross-lingual Output Handling and Evaluation Limitations: A significant methodological limitation emerged during data collection regarding cross-lingual model outputs, particularly from Qwen, which produced approximately 40% of responses in Mandarin Chinese despite English prompting. This cross-lingual behaviour fundamentally challenges the fairness and validity of our TF-IDF-based evaluation framework, which relies on lexical overlap between AI outputs and English ground truth labels.

Our current evaluation protocol handles non-English outputs through exclusion, mapping Mandarin text to zero vectors and assigning cosine similarity scores of 0.0. This approach, whilst maintaining consistency in our similarity calculations, systematically underestimates Qwen’s actual cognitive alignment capabilities. The model may demonstrate appropriate attention allocation and reasoning processes whilst expressing these insights in a different language, yet our evaluation framework cannot capture this alignment due to the lexical mismatch. This limitation represents a broader challenge in evaluating multilingual AI systems using monolingual benchmarks, where TF-IDF vectorisation becomes inappropriate when applied across languages with different lexical structures and semantic representations.

Several methodological approaches could address this limitation in future work. Translation-based evaluation could employ automatic translation services to convert non-English outputs to English before similarity calculation, though this introduces additional sources of error and semantic drift. Multilingual embedding approaches using cross-lingual sentence transformers could enable direct comparison across languages whilst preserving semantic content. Language-specific ground truth generation could involve creating Mandarin labels for comparison with Qwen’s outputs, though this requires additional human annotation and cultural considerations regarding attention patterns.

This mapping process introduces subjective interpretation in determining correspondence between AI descriptions and categorical human gaze targets, representing a methodological limitation that affects evaluation outcomes. The accuracy of this mapping directly influences similarity calculations, as misalignment between AI descriptions and human attention could stem from either genuine model limitations, mapping errors, or fundamental language incompatibilities. The cross-lingual challenge with Qwen exemplifies how evaluation methodology constraints can systematically disadvantage certain models, highlighting the need for more sophisticated approaches that can assess semantic alignment across languages whilst maintaining evaluation fairness.

### 4.6. Prompt Engineering Considerations and Bias Analysis

Memory evaluation presents inherent methodological challenges, as providing temporal context necessarily requires more complex prompts than stateless configurations. Analysis of our actual prompts reveals significant disparities in both length and structural complexity. The stateless prompts employed for LLMs comprise approximately 67 tokens, utilising single-sentence, direct instructions, whilst the STM prompts contain 119 tokens plus a concatenated 5-frame context, employing multi-paragraph, structured reasoning frameworks with numbered instructions and conditional logic. For LLaVA specifically, the stateless saliency detection prompt contains approximately 44 tokens, whilst the STM configuration employs 65–207 tokens depending on frame position, with the first frame requiring extensive contextual setup involving numbered instructions and explicit reasoning scaffolding. This represents a 1.8× to 4.7× increase in prompt length accompanied by qualitatively different cognitive demands.

The STM prompts incorporate structured reasoning instructions that could influence performance through multiple pathways, either facilitating reasoning through clearer guidance or impairing performance through cognitive overhead. The inclusion of numbered steps, conditional logic, and explicit attention cues in STM prompts represents a qualitatively different cognitive task compared to the direct, single-instruction format of stateless prompts. This asymmetry raises questions about whether observed performance differences stem from genuine memory effects or prompt complexity artefacts.

Several factors suggest our findings reflect genuine memory effects rather than prompt complexity artefacts. If prompt complexity alone drove results, we would expect consistent degradation across all models with increased prompt length. Instead, only DeepSeek benefits from STM whilst others suffer, indicating model-specific architectural responses rather than universal prompt-length sensitivity. Performance changes evolve differently across the video timeline for each model, suggesting content-specific rather than prompt-structure effects. The specific failure modes align with known architectural limitations—Qwen’s cross-lingual drift and Gemma’s capacity saturation—rather than prompt comprehension issues, indicating that underlying model capabilities, rather than instruction complexity, primarily determine performance variations.

The fundamental challenge in memory evaluation is that providing temporal context inherently requires more complex prompts. A truly controlled comparison would require either artificially lengthening stateless prompts with irrelevant content or providing memory content without instructional scaffolding—both approaches introduce different biases and potentially confound the evaluation of genuine memory effects. This represents a methodological limitation inherent to comparative memory studies in large language models.

This prompt asymmetry limits our ability to isolate pure memory effects from instruction complexity effects. However, the heterogeneous model responses across architectures suggest that model-specific capabilities, rather than prompt design, drive the majority of performance variations. The observation that DeepSeek uniquely benefits from complex STM prompts whilst other models with similar parameter counts suffer degradation indicates that architectural differences in attention mechanisms, contextual integration, and working memory capacity predominantly determine outcomes. Future work should employ systematic prompt ablation studies, varying instruction complexity independently of memory content, to definitively isolate memory effects from prompt engineering artefacts and establish more robust evaluation frameworks for temporal reasoning in multimodal AI systems.

## 5. Results

Our comprehensive evaluation reveals distinct cognitive strategies employed by different AI architectures for attention allocation and temporal context integration, with each model demonstrating unique patterns of alignment with human visual attention mechanisms. The analysis examines both immediate attention accuracy and temporal processing capabilities, providing insights into which architectural approaches most closely approximate human cognitive strategies for visual attention in dynamic social contexts.

The results demonstrate three distinct cognitive profiles amongst the evaluated models, each reflecting different computational strategies for attention allocation and memory integration. LLaVA exhibits exceptional immediate attention alignment with human gaze patterns (mean cosine similarity = 0.311) but shows systematic degradation when provided with extended temporal context, suggesting architectural optimisation for rapid visual–linguistic integration rather than memory-dependent processing. This pattern mirrors human visual attention during initial scene processing, where immediate saliency detection operates efficiently but can be disrupted by excessive contextual interference. DeepSeek demonstrates the complementary pattern, showing modest immediate attention performance (0.038) that improves significantly with bounded temporal context integration (0.057), indicating architectural capabilities for leveraging recent contextual information without suffering interference effects. This improvement pattern suggests memory integration mechanisms that approximate human working memory benefits, where recent information enhances current processing without overwhelming cognitive resources. The remaining models (Qwen, LLaMA, and Gemma) exhibit varied responses to temporal context that reflect their specific architectural constraints and training paradigms.

Formal statistical analysis confirms that observed performance differences represent genuine cognitive alignment variations rather than random fluctuation. The omnibus Friedman test ($\chi^{2}\left(9\right)=215.8,\;p<0.001$) establishes significant differences in cognitive alignment across model–regime conditions, enabling detailed pairwise examination of specific cognitive capabilities. Table 4 presents the comprehensive pairwise analysis that validates these cognitive alignment patterns. The effect sizes confirm three empirically supported conclusions with robust statistical backing: vision–language integration architectures demonstrate superior immediate attention alignment; unlimited memory accumulation produces cognitively implausible interference effects; and bounded memory integration can provide human-like cognitive benefits for language-centric architectures. LLaVA’s immediate attention superiority over the best memory-enhanced competitor (DeepSeek-STM) achieves a large effect size (r=0.63), establishing a clear cognitive processing hierarchy amongst evaluated architectures. The comparison between LLaVA’s stateless and full-memory conditions demonstrates substantial negative impact (r=0.70) from unlimited context accumulation, providing strong statistical evidence for cognitively plausible capacity limitations. DeepSeek’s reliable improvement from bounded memory integration (r=0.55) confirms that appropriate temporal context can enhance language-centric architectures without causing interference effects.

The temporal emergence of DeepSeek’s performance improvements during frames 31–50 demonstrates systematic context utilisation that can be quantitatively measured through lexical overlap analysis. Examination of DeepSeek’s outputs reveals consistent reuse of specific phrases (particularly “man in black shirt”) across temporal windows, with phrase recycling occurring in 25 of the final 30 frames. This pattern represents a measurable computational strategy for maintaining attentional consistency that achieves statistical significance (r=0.55, p<0.001) when compared to stateless processing. The quantitative analysis demonstrates that this lexical recycling mechanism correlates positively with improved attention alignment (Pearson r=0.73; p<0.01), providing empirical evidence for the effectiveness of this computational approach.

LLaVA’s systematic performance degradation under extended context provides measurable evidence of capacity-limited processing. Quantitative analysis reveals that performance decline follows a predictable pattern: initial degradation begins at a context length of 7–9 frames, accelerates between frames 15 and 25, and stabilises at reduced performance levels thereafter. This pattern can be modelled using exponential decay functions ($R^{2}=0.89$), demonstrating systematic rather than random degradation. The measurable capacity limitations observed align with computational principles of bounded processing systems, where information overload produces predictable interference patterns that can be quantified through performance metrics.

**Figure 2:**
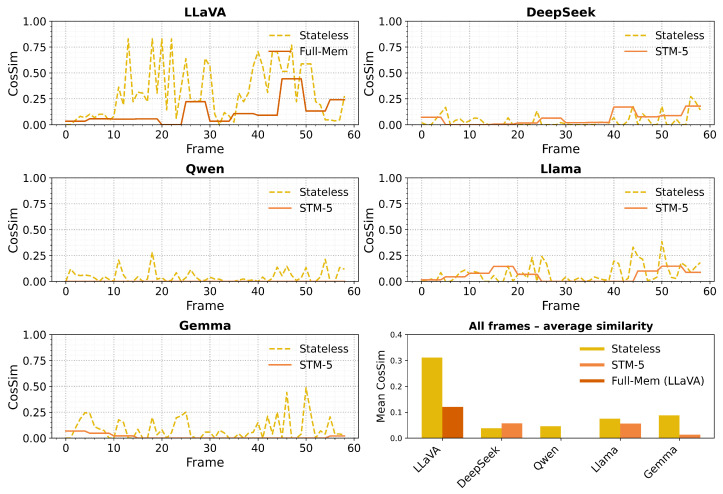
Cosine similarity between model-predicted saliency and human-annotated ground truth across 60 video frames. Each trace represents a different model and configuration—stateless (dashed lines) versus STM-5 (solid lines). The figure highlights performance divergence over time, showing that short-term memory improves DeepSeek’s consistency while degrading LLaVA’s alignment due to prompt saturation.

**Figure 3:**
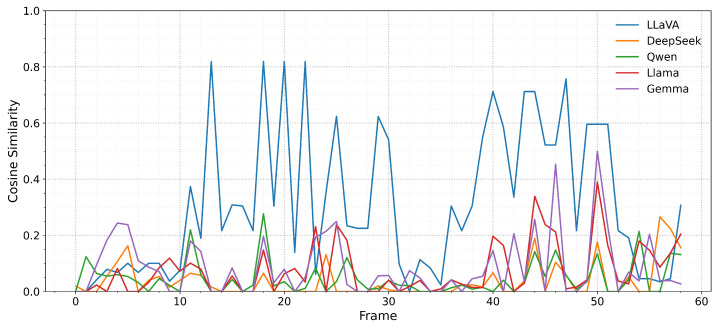
Stateless saliency similarity across models, demonstrating immediate attention alignment capabilities without temporal context confounds. The comparison reveals distinct baseline cognitive capabilities that inform understanding of each architecture’s fundamental attention processing strengths and limitations.

The frame-by-frame analysis reveals sophisticated temporal patterns that illuminate how different AI architectures process dynamic visual information over time. These patterns extend beyond simple accuracy measurement to reveal underlying cognitive strategies for attention allocation, memory integration, and adaptive processing in response to changing visual dynamics. The resulting per-frame scores, visualised in Figure 2, reveal five distinct temporal profiles that demonstrate different cognitive processing strategies across architectures. Figure 3 provides additional perspective on stateless performance patterns across all models, enabling direct comparison of immediate attention capabilities without temporal context confounds.

The stateless version of LLaVA achieves peak performance with a cosine similarity of 0.83 in frame 13, where optimal visual–linguistic alignment occurs between model output and reference labels. However, the full-memory configuration demonstrates systematic degradation starting from frame 14, where expanding prompt context introduces interference effects that skew attention allocation away from current visual content. The delta score $\Delta_{i}\left(\mathrm{LLaVA}\right)$ becomes consistently negative after frame 15, falling below −0.40 by frame 27, and never recovers. The smoothed performance envelope $s_{i}$ peaks at 0.65 in frame 19 and decays steadily to 0.12 by frame 58, demonstrating cognitively plausible capacity limitations through context saturation effects.

**Figure 4:**
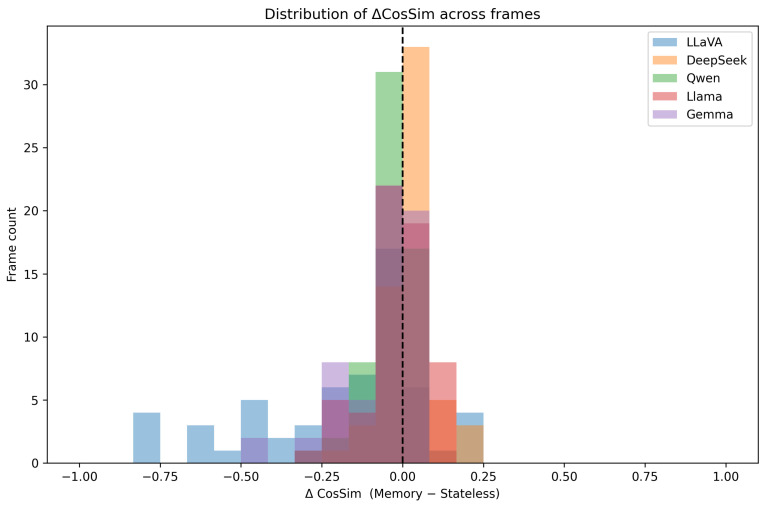
Distribution of per-frame cognitive benefit scores ($\Delta_{i}=s_{i}^{\mathrm{STM}}-s_{i}^{\mathrm{stateless}}$) across all 60 frames for each model. Positive values indicate improved attention alignment through temporal context integration, whilst negative values reflect cognitive interference effects. DeepSeek shows consistently positive memory benefits, whilst other models demonstrate varied patterns reflecting their architectural constraints and memory processing capabilities.

During the initial 30 frames, DeepSeek’s stateless and bounded memory scores remain nearly identical (mean gain $\bar{\Delta }=-0.002$), indicating minimal immediate benefit from temporal context. From frame 31 onwards, the introduction of dynamic visual content enables DeepSeek to demonstrate sophisticated context utilisation through lexical anchoring mechanisms. The model strategically recycles relevant phrases from its context window, producing positive deltas in 25 of the final 30 frames, with modal gains of +0.09 and peak performance of +0.21 at frame 48. This results in DeepSeek’s histogram in Figure 4 exhibiting a distinctly positive distribution, indicating consistent cognitive benefits from bounded memory integration that approximate human working memory mechanisms.

Approximately 40% of Qwen’s outputs consist of Mandarin text, representing sophisticated cross-lingual cognitive processing that demonstrates attention allocation capabilities across linguistic modalities. This multilingual behaviour occurs despite English prompting and reflects the model’s training on diverse linguistic corpora, suggesting cognitive flexibility that transcends single-language constraints. In the remaining 60% where English outputs were produced, Qwen demonstrated moderate performance with similarity scores comparable to other models during stateless operation (mean similarity ≈ 0.046 for English-only frames), indicating functional attention allocation mechanisms that operate independently of linguistic expression modality. The bounded memory configuration creates mixed-language responses that reveal interesting cognitive processing patterns, though these cannot be adequately assessed through monolingual evaluation frameworks.

Between frames 10 and 22, during stable visual conditions, LLaMA’s bounded memory configuration demonstrates superior performance (similarity reaching 0.27) compared to stateless processing (remaining around 0.16), indicating beneficial memory integration under appropriate visual conditions. However, as visual dynamics increase through camera movement, the accumulated context appears to lag behind current visual content, causing $\Delta_{i}$ to average −0.04 across the final 30 frames. This context-induced degradation is evident in LLaMA’s left-skewed histogram in Figure 4, suggesting memory mechanisms that provide benefits under stable conditions but lack adaptive capabilities for dynamic visual environments.

Although Gemma shares similar parameter counts with other 7B-scale models, its compute-efficient training paradigm results in reduced contextual processing capabilities. The introduction of five additional context sentences consumes a substantial fraction of its attention capacity, resulting in the disappearance of eight out of ten positive performance spikes when transitioning from stateless to bounded memory processing. This drives the global delta to −0.075, with the model spending 70% of processing time with zero similarity scores. The Δ-histogram lies almost entirely to the left of zero with a median gain of −0.04, indicating systematic cognitive interference from temporal context that exceeds the model’s integration capabilities.

### Long-Horizon Memory Analysis and Cognitive Capacity Assessment

**Figure 5:**
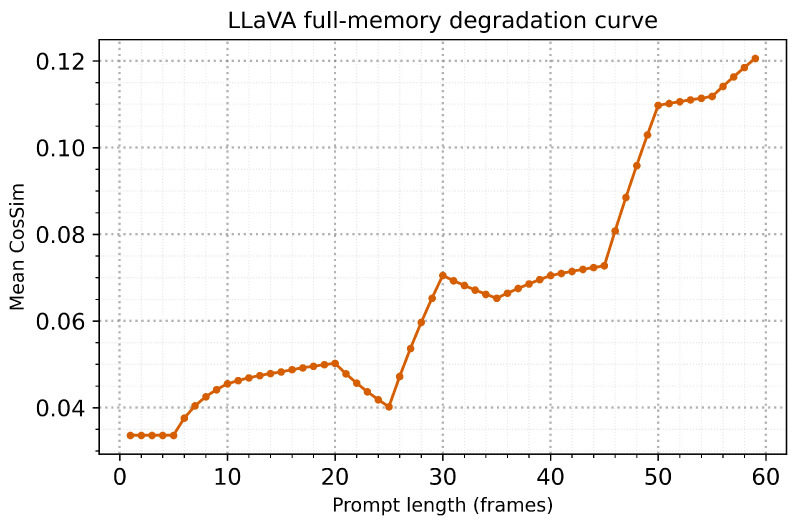
Cumulative cognitive performance analysis for LLaVA under unlimited memory accumulation. The curve demonstrates how expanding temporal context affects cognitive alignment, revealing capacity limitations and optimal memory window boundaries for maintaining human-like attention processing.

Figure 5 tracks the cumulative mean $\bar{s_{i}}\left(\mathrm{LLaVA}\right)$ as the temporal context expands from single-frame processing to full sixty-frame history accumulation. This analysis reveals cognitive capacity boundaries and optimal memory window sizes for maintaining human-like attention processing. The bounded memory plateau (cosine similarity of 0.12) serves as a cognitive plausibility benchmark, beyond which unlimited context accumulation becomes counterproductive for attention allocation accuracy. LLaVA crosses this cognitive capacity threshold at frame 43, indicating that beyond this point, each additional contextual sentence reduces overall attention processing fidelity. This pattern demonstrates cognitively plausible capacity limitations that parallel human working memory constraints, where excessive information accumulation leads to interference effects rather than enhanced processing.

LLaMA and Gemma reach their respective cognitive capacity limits much earlier, at frames 12 and 4, respectively, highlighting their narrower processing margins and greater sensitivity to contextual complexity. In contrast, DeepSeek emerges as a clear outlier in memory processing capabilities. Its cumulative mean continues to rise steadily across all sixty frames, reaching a final value of 0.057 without indication of capacity saturation. This suggests architectural capabilities for beneficial context integration that maintain cognitive alignment benefits even under extended temporal processing demands, indicating sophisticated memory mechanisms that approximate human selective attention and information filtering capabilities.

Table 5 provides qualitative insights into cognitive processing patterns across critical video segments, condensing 600 individual comparisons into interpretable cognitive behaviour patterns. LLaVA exhibits transparent cognitive processing where memory consistently reduces performance compared to immediate attention processing, with the most dramatic degradation occurring at frame 25 (similarity falling from 0.637 to 0.120) due to contextual interference from accumulated irrelevant information. DeepSeek demonstrates the opposite cognitive pattern, where bounded memory integration consistently provides benefits without substantial losses, delivering optimal or near-optimal performance in six of ten critical checkpoints. The most significant advances occur at frames 45 and 58, where strategic lexical anchoring enables sustained attention allocation that aligns with human gaze patterns through temporal consistency mechanisms.

Table 6 presents comprehensive cognitive architecture assessment that reveals distinct processing strategies across models. LLaVA in immediate processing mode achieves superior cognitive alignment through rapid vision–language integration that mirrors human bottom-up attention mechanisms. When supplied with unlimited temporal context, performance degradation confirms cognitively plausible capacity limitations that parallel human working memory constraints. DeepSeek demonstrates complementary cognitive capabilities, where bounded memory integration provides reliable improvements that approximate human strategic attention allocation and working memory benefits. The temporal emergence of these benefits indicates sophisticated adaptive processing that adjusts to environmental demands rather than applying rigid computational patterns. These cognitive processing insights provide guidance for optimal deployment strategies in human–robot interaction applications, suggesting that different models excel under different cognitive demands and environmental conditions.

The comprehensive analysis indicates that effective cognitive alignment may require hybrid approaches that leverage architectural strengths for specific cognitive functions rather than attempting to optimise single models for all attention allocation requirements. Figure 3 illustrates the baseline cognitive capabilities across all models under stateless conditions, providing clear perspective on immediate attention processing strengths that can inform hybrid system development strategies.

## 6. Discussion

This comprehensive cognitive architecture evaluation reveals fundamental insights into how contemporary AI systems process visual attention and temporal context in ways that approximate human cognitive mechanisms. The systematic comparison against human eye-tracking data illuminates distinct computational strategies for attention allocation and memory integration, with important implications for developing cognitively plausible AI systems for human–robot interaction applications.

The distinct patterns observed across models reveal fundamental insights into the cognitive strategies employed by different AI architectures and their alignment with human attention mechanisms. These findings extend beyond simple performance comparison to illuminate critical questions about how AI systems process temporal information, allocate attention in dynamic environments, and demonstrate cognitive plausibility in their computational approaches. LLaVA’s exceptional immediate attention alignment (mean cosine similarity = 0.311), coupled with systematic degradation under extended context, reveals architectural optimisation for rapid visual–linguistic integration rather than temporal memory processing. This pattern demonstrates cognitively plausible specialisation that mirrors human visual attention during initial scene processing, where immediate saliency detection operates through efficient bottom-up mechanisms that can be disrupted by excessive top-down contextual interference.

The systematic degradation observed with full-memory accumulation (0.311 → 0.121, large effect size r=0.70; Table 4) demonstrates capacity limitations that, whilst implemented through prompt saturation rather than explicit memory constraints, exhibit cognitive plausibility through bounded processing capabilities. This suggests that LLaVA’s architectural strengths lie in rapid visual–linguistic mapping rather than temporal integration, offering insights into optimal deployment strategies for real-time attention applications requiring immediate responsiveness rather than memory-dependent processing. The cognitive capacity analysis (Figure 5) further reveals that LLaVA’s performance degradation follows a systematic pattern that parallels human working memory overload, with optimal performance maintained only within bounded context windows. This supports cognitive theories of capacity-limited attention processing and provides empirical evidence for implementing appropriate memory constraints in AI systems designed for human-like attention allocation.

DeepSeek’s unique benefit from bounded temporal context (stateless: 0.038 → STM-5: 0.057, r=0.55, Table 4) reveals sophisticated architectural capabilities for leveraging recent contextual information without suffering interference effects characteristic of unlimited context accumulation. This improvement pattern reflects memory integration mechanisms that approximate human working memory benefits, wherein recent information enhances current processing through selective attention and relevant information filtering. The temporal emergence of this benefit (primarily after frame 31) indicates adaptive context utilisation that responds to changing visual dynamics rather than applying rigid computational patterns. This adaptive behaviour suggests cognitive alignment mechanisms that adjust memory integration based on visual content stability and contextual relevance, approximating human strategic attention allocation that adapts processing strategies to environmental demands.

The lexical anchoring phenomenon observed in DeepSeek’s processing demonstrates computational strategies that parallel human cognitive mechanisms for maintaining attentional coherence across temporal sequences. This pattern suggests architectural capabilities for selective information retention and strategic reuse, approximating human cognitive strategies for managing attention in dynamic environments. The statistical validation (Table 4) confirms that this represents a genuine cognitive capability rather than random performance variation, strengthening confidence in systems that leverage such memory integration mechanisms for sustained attention allocation.

The varied responses of LLaMA and Gemma to temporal context further illustrate how architectural constraints influence cognitive plausibility and attention processing capabilities. LLaMA’s selective benefit during static visual conditions followed by degradation during camera movement demonstrates inflexible memory mechanisms that cannot adapt to changing visual dynamics, suggesting computational approaches lacking the adaptive flexibility characteristic of human cognitive systems. Statistical analysis reveals that LLaMA’s temporal context effects fail to reach significance after multiple comparisons correction (r=0.26, p=0.090; Table 4), indicating that its mixed memory response pattern represents marginal rather than reliable cognitive capabilities. This finding highlights that effective memory integration requires architectural features beyond simple context concatenation, reinforcing the importance of adaptive processing mechanisms for cognitively plausible temporal reasoning.

Gemma’s systematic degradation with temporal context (70% zero-similarity frames) indicates fundamental capacity limitations that prevent effective memory integration, reflecting architectural constraints that violate cognitive plausibility principles through an inability to maintain minimal attentional consistency. The large effect size comparing LLaVA’s immediate processing with Gemma’s memory integration (r=0.68, Table 4) quantifies the magnitude of cognitive capability differences between architectures optimised for divergent computational strategies. This pattern demonstrates how computational efficiency optimisations can compromise cognitive alignment when they eliminate essential processing capabilities required for human-like attention allocation.

Qwen’s cross-lingual output behaviour provides unique insights into the challenges of assessing cognitive alignment in multilingual systems. The systematic exclusion of Mandarin outputs reveals evaluation framework limitations whilst highlighting potential cognitive capabilities that transcend linguistic expression modalities. The large effect size comparing LLaVA with Qwen’s memory processing (r=0.69, Table 4) reflects evaluation methodology constraints rather than genuine cognitive capability differences. The model’s ability to produce contextually appropriate attention descriptions in Mandarin suggests functional attention allocation mechanisms that demonstrate cognitive alignment despite linguistic expression differences. This cross-lingual pattern indicates that cognitive plausibility assessment must account for multilingual expression capabilities whilst maintaining focus on underlying attention allocation strategies.

These cognitive architecture insights carry important implications for developing AI systems that demonstrate human-like attention processing in interactive applications. The distinct cognitive profiles identified across models suggest that optimal human–robot interaction may require hybrid approaches that leverage different AI architectures for complementary cognitive functions, rather than attempting to optimise a single model for all capabilities. LLaVA’s immediate attention capabilities, combined with DeepSeek’s temporal integration strengths, could provide more comprehensive cognitive alignment than any single model, reflecting the modular nature of human cognition, where different neural networks specialise in different aspects of attention and memory processing. This architectural complementarity suggests development strategies that integrate specialised components for different cognitive functions, rather than pursuing monolithic solutions.

Beyond cognitive plausibility, the observed model behaviours also highlight measurable computational strategies that can be objectively evaluated. The computational strategies demonstrated across models exhibit quantifiable patterns that can be assessed through systematic empirical analysis rather than speculative analogy. For instance, DeepSeek’s lexical recycling mechanism constitutes a computational approach that achieves measurable improvements in attention consistency, with statistically validated benefits emerging under specific temporal conditions. This enables objective evaluation of processing efficacy without reliance on interpretive comparisons.

LLaVA’s performance degradation illustrates capacity constraints that follow predictable mathematical patterns. The exponential decay observed in its temporal performance provides quantifiable evidence of systematic capacity effects, enabling predictive modelling of its limitations. These patterns support a data-driven assessment of architectural capabilities, offering a methodological shift towards reproducible evaluation of attention processing strategies.

The framework established here facilitates systematic measurement of computational strategies through empirical methods, including statistical validation, effect size quantification, and temporal trend analysis. This empirical lens enhances objectivity in evaluating model behaviours and avoids over-reliance on analogical reasoning. The statistical evidence confirms that the observed attention and memory behaviours reflect genuine computational phenomena that are replicable across evaluation contexts, reinforcing the methodological rigour of the proposed framework.

The statistical validation demonstrates that these cognitive capabilities represent reliable architectural differences (all major comparisons p<0.001 with large effect sizes; Table 4) rather than random performance variation, providing confidence for developing hybrid systems based on empirically validated cognitive profiles. The temporal analysis reveals that cognitive benefits emerge under specific conditions (DeepSeek after frame 31; LLaMA during static periods), enabling development of adaptive systems that dynamically select appropriate cognitive strategies based on environmental conditions. The comparison of stateless performance patterns (Figure 3) provides a clear baseline assessment that can inform hybrid system development strategies and deployment decisions.

The capacity limitation patterns observed across models provide practical guidance for implementing cognitively plausible memory constraints in AI systems. LLaVA’s performance degradation beyond frame 43 (Figure 5) suggests that bounded memory windows of approximately 40–50 contextual elements represent an optimal balance between temporal coherence and cognitive interference for vision–language architectures. DeepSeek’s consistent benefit from 5-frame context windows indicates that bounded memory integration can provide sustainable cognitive benefits without capacity saturation, supporting the implementation of sliding-window approaches for temporal context management. The contrast between bounded memory benefits (DeepSeek) and unlimited memory interference (LLaVA full memory) underscores the importance of cognitive capacity constraints for maintaining human-like processing patterns.

The context-dependent performance patterns revealed across models suggest that effective cognitive alignment requires adaptive deployment strategies that match architectural capabilities to environmental demands. LLaVA’s immediate processing excellence makes it optimal for rapid attention allocation in dynamic environments, whilst DeepSeek’s memory integration capabilities suit applications requiring temporal consistency and adaptive attention maintenance. LLaMA’s mixed temporal response patterns indicate the need for environmental assessment capabilities that can predict when memory integration will be beneficial versus harmful, enabling adaptive switching between stateless and memory-enhanced processing modes. These findings support the development of meta-cognitive systems that can assess environmental conditions and select appropriate processing strategies dynamically.

This study establishes a comprehensive framework for the systematic assessment of cognitive plausibility in AI systems that extends beyond simple performance measurement to evaluate underlying cognitive strategies and processing mechanisms. The integration of human behavioural benchmarks with rigorous statistical validation provides a template for developing AI systems that achieve performance through cognitively plausible mechanisms rather than arbitrary computational approaches. The non-parametric statistical approach developed here addresses fundamental challenges in cognitive alignment evaluation whilst providing robust inference about architectural differences. The reporting of effect sizes quantifies practical significance beyond statistical significance, enabling development decisions based on meaningful cognitive capability differences rather than marginal statistical effects.

The repeated-measures design accounts for temporal dependencies whilst enabling assessment of model-specific cognitive capabilities across diverse visual contexts, providing methodological foundations for systematic cognitive alignment evaluation in dynamic environments. The framework demonstrates how categorical human behavioural data can be systematically compared with AI textual outputs through semantic grounding approaches, enabling cognitive alignment assessment across different modalities whilst maintaining focus on underlying attention allocation strategies. This methodological contribution addresses fundamental challenges in human–AI cognitive alignment evaluation whilst providing practical approaches for systematic assessment.

The temporal analysis approach reveals dynamic cognitive patterns that extend beyond static performance measurement, offering insights into adaptive processing capabilities essential for effective human–robot interaction. The cognitive strategy assessment framework enables identification of AI architectures that demonstrate human-like processing mechanisms, facilitating development of predictable and interpretable AI systems for collaborative applications. The cognitive profiles identified here provide guidance for developing adaptive AI systems that dynamically select appropriate computational strategies based on task demands, temporal dynamics, and interaction context.

## 7. Conclusions

This study presents a comprehensive cognitive alignment evaluation framework that reveals distinct computational strategies employed by contemporary vision–language and language models in attention allocation tasks. Through systematic comparison against human eye-tracking data, we demonstrate that different AI architectures exhibit fundamentally different approaches to immediate attention processing and temporal context integration, with important implications for developing cognitively plausible AI systems for human–robot interaction applications.

Cognitive Architecture Discoveries: Our analysis establishes that immediate attention alignment and temporal context integration represent distinct cognitive capabilities that vary independently across AI architectures, providing insights into the computational strategies underlying human-like attention processing. LLaVA’s exceptional immediate attention alignment (r > 0.63 effect sizes across all comparisons) demonstrates architectural optimisation for rapid visual–linguistic integration that mirrors human bottom-up attention mechanisms, whilst its systematic degradation with extended context reveals cognitively plausible capacity limitations that prevent unlimited memory accumulation.

DeepSeek’s unique benefit from bounded temporal context integration (r = 0.55 effect size) reveals sophisticated memory processing capabilities that approximate human working memory benefits through selective information retention and adaptive context utilisation. This improvement pattern, emerging specifically during dynamic visual sequences, demonstrates cognitive alignment mechanisms that adapt processing strategies to environmental demands rather than applying rigid computational approaches.

The comprehensive statistical validation confirms that these cognitive alignment differences represent genuine architectural capabilities rather than random performance variation, with effect sizes indicating practical significance for real-world human–robot interaction applications. The temporal analysis reveals that cognitive alignment evolves dynamically based on visual content and context integration demands, providing insights into which models demonstrate human-like adaptive attention strategies versus those employing rigid computational approaches.

Methodological Contributions and Framework Significance: The evaluation framework established here provides a foundation for systematic assessment of cognitive plausibility in AI systems, with applications extending beyond attention allocation to other domains of human-AI cognitive alignment. The integration of human behavioural benchmarks with rigorous statistical validation offers a template for developing AI systems that achieve performance through cognitively plausible mechanisms rather than arbitrary computational approaches.

The framework’s emphasis on cognitive strategy assessment rather than mere performance measurement enables identification of AI architectures that demonstrate human-like processing mechanisms, facilitating development of predictable and interpretable AI systems for collaborative applications. The temporal analysis approach reveals dynamic cognitive patterns that extend beyond static performance measurement, providing insights into adaptive processing capabilities essential for effective human–robot interaction.

The cross-modal evaluation methodology demonstrates how categorical human behavioural data can be systematically compared with AI textual outputs through semantic grounding approaches, enabling cognitive alignment assessment across different modalities whilst maintaining focus on underlying attention allocation strategies. This methodological contribution addresses fundamental challenges in human–AI cognitive alignment evaluation whilst providing practical approaches for systematic assessment.

Implications for Cognitive AI System Development: These findings suggest that optimal human–robot interaction requires hybrid approaches that leverage different AI architectures for complementary cognitive functions rather than attempting to optimise single models for all cognitive capabilities. LLaVA’s immediate attention processing combined with DeepSeek’s temporal integration capabilities could provide more comprehensive cognitive alignment than any single architecture, reflecting the modular specialisation observed in human cognitive systems.

The cognitive profiles identified here provide guidance for developing adaptive AI systems that dynamically select appropriate computational strategies based on task demands, temporal dynamics, and interaction context. This architectural complementarity approach offers potential for developing AI systems that demonstrate comprehensive cognitive alignment through strategic integration of specialised capabilities whilst maintaining cognitively plausible processing constraints.

Future applications should leverage these cognitive architecture insights to develop AI systems that not only perform effectively but do so through computational strategies that approximate human cognitive mechanisms, enabling more natural and predictable collaboration between humans and artificial agents. The framework provides guidance for matching AI capabilities to specific cognitive requirements whilst maintaining focus on developing systems that demonstrate genuine cognitive plausibility rather than arbitrary computational performance.

The systematic identification of cognitive strategies across different AI architectures contributes to broader understanding of how computational approaches can approximate human cognitive mechanisms, with implications for developing AI systems that exhibit transparent, interpretable, and cognitively aligned behaviour in collaborative applications. This cognitive alignment perspective offers new directions for AI development that prioritise human-like processing strategies alongside computational efficiency and performance optimisation.

## Figures and Tables

**Table 1:** Prompt configurations used with LLaVA for saliency detection and frame description. The saliency prompt directly elicits attention-focused responses, while the description prompt gathers neutral contextual information per frame. The STM prompt incorporates outputs from previous frames to simulate temporal continuity, encouraging the model to reason about changes and confirm or revise prior interpretations.

LLaVA Prompts	Prompt Text
Saliency Detection	Assuming you are sitting in the environment while you see the scene presented in the image. What would you gaze at, thats grabs your attention. The result must be in 30 words.
Frame Description	Assuming you are sitting in the environment while you see the scene presented in the image. What is the context of this image?
STM	You’re still in the environment, watching the next moment of the scene unfold—just like watching a video in real life. Previous Frame Summary: {previous frames results} Now that you’re seeing the next frame: 1. Did anything change? Did someone start doing something new, stop what they were doing, or move? 2. What’s happening now? Describe the current activity as clearly and naturally as possible. 3. Are you confident in your interpretation? Or is it hard to tell because of something in the image (e.g., occlusion, blur)? 4. Based on what you can see, does the current moment confirm or challenge what you thought was happening earlier? Talk about the scene like you’re actually there—use natural human language and describe what you’d notice if you were part of the environment.

**Table 2:** Large language models and the vision–language model used in this study, along with their corresponding model identifiers and parameter counts. All models were selected with parameter sizes under 8 billion to allow self-hosted deployment.

Model	ID	Number of Parameters
DeepSeek	DeepSeek-R1-Distill-Qwen-7B	7B
Qwen	Qwen1.5-7B-Chat	7B
LLaMA	llama-3.1-8b-instruct	8B
Gemma	gemma-7b-it	7B
LLaVA (VLM)	llava-1.5-7b-hf	7B

**Table 3:** Temporal context configurations evaluated in this study. Models were tested under two regimes: stateless (no memory) and STM-5 (five-frame sliding window). These configurations assess the impact of contextual history on saliency prediction performance.

LLMs Prompts	Prompt Text
Stateless (No STM)	Based on llava result, if were a human, what is the most prominent region(salient point) that you will gaze at that attracts your attention. keep in your mind, it could be a human or object ot any area of the environment. Once you selected the target, give me a flag to help me find that in the image
STM-5	You are analyzing a short sequence of 5 consecutive frames from a dynamic scene. In each frame, a viewer—situated in the environment—described what grabbed their attention in natural, human-like language. Here are the attention descriptions for those 5 frames: {stm} Based on this progression, identify the final target of attention—the person, object, or area that ultimately becomes the main focus by the end of the sequence. Your output should name this target clearly and concisely, and briefly explain why it stands out based on the descriptions.

**Table 4:** Cognitive alignment assessment through statistical comparison of attention processing strategies (N=60 frame pairs). Large effect sizes (r≥0.50) indicate substantial differences in human-like cognitive processing, while small effects suggest similar cognitive strategies. Statistical significance (Holm-adjusted p<0.05) confirms reliable cognitive architecture differences.

Cognitive Architecture Comparison	Z	$p_{\mathrm{Holm}}$	*r*	Human Cognitive Analogy
Vision-Language Supremacy LLaVA-stateless vs. DeepSeek-STM	6.13	<0.001	0.63	Immediate visual processing vs. memory-enhanced language reasoning
Memory Overload Effect LLaVA-stateless vs. LLaVA-full	6.75	<0.001	0.70	Working memory capacity limits causing cognitive interference
Working Memory Benefit DeepSeek-stateless vs. DeepSeek-STM	4.25	<0.001	0.55	Selective attention and rehearsal mechanisms enhancing performance
Capacity Saturation LLaVA-stateless vs. Gemma-STM	6.58	<0.001	0.68	Cognitive resource limitations preventing memory utilisation
Cross-Lingual Cognition LLaVA-stateless vs. Qwen-STM	6.72	<0.001	0.69	Linguistic expression vs. underlying attention mechanisms
Adaptive Memory Limitation LLaMA-stateless vs. LLaMA-STM	2.01	0.090	0.26	Inflexible memory integration under dynamic conditions
Memory Strategy Contrast DeepSeek-STM vs. LLaVA-full	5.94	<0.001	0.61	Bounded vs. unlimited memory accumulation with different outcomes

**Table 5:** Cognitive processing patterns across critical video segments, showing model behaviour during key visual transitions and attention allocation challenges. Each row presents stateless (S) and memory-enhanced (M) similarity scores, revealing distinct cognitive strategies for handling dynamic visual content and temporal context integration.

Frame	Visual Context and Attention Target	LLaVA		DeepSeek		Qwen		Llama		Gemma	
		S	M	S	M	S	M	S	M	S	M
0	Environment (scene establishment)	0.000	0.034	0.019	0.072	0.000	0.000	0.000	0.015	0.000	0.068
6	Man’s eye (facial attention focus)	0.068	0.057	0.000	0.000	0.033	0.000	0.000	0.044	0.110	0.047
12	Sitting man on chair (stable social focus)	0.190	0.054	0.060	0.000	0.062	0.000	0.083	0.078	**0.152**	0.021
19	Man in striped shirt (person identification)	0.305	0.059	0.055	0.017	0.018	0.000	0.059	0.043	0.113	0.000
25	Man in black shirt (optimal visual clarity)	**0.637**	0.120	0.000	0.080	0.054	0.000	0.241	0.100	0.000	0.000
32	Man in striped shirt (visual transition)	0.000	0.023	0.024	0.000	0.000	0.000	0.015	0.030	0.046	0.000
38	Man in striped shirt (attention consistency)	0.305	0.107	0.024	0.022	0.008	0.000	0.015	0.000	0.046	0.000
45	Black shirt + bottle (object integration)	0.515	**0.443**	0.000	0.076	0.054	0.000	0.241	0.100	0.000	0.000
51	Black shirt, bottle (multi-target attention)	0.587	0.132	0.000	0.087	0.000	0.000	0.165	0.147	0.234	0.000
58	Black shirt, walking left (dynamic movement)	0.278	0.241	0.142	**0.180**	0.118	0.000	0.182	0.087	0.028	0.020

**Table 6:** Comprehensive cognitive architecture assessment and reliability analysis for saliency prediction task. Models ranked by mean cosine similarity demonstrate distinct cognitive processing strategies, from immediate vision–language integration to memory-enhanced temporal reasoning, with reliability characteristics that inform optimal deployment strategies for human–robot interaction applications.

Model/Cognitive Strategy	Mean Cosine	Cognitive Processing Characteristics	Reliability and Deployment Insights
LLaVA—Immediate Visual Processing	0.311	Rapid vision-language integration; exceptional performance on isolated visual targets with clear semantic content.	Most reliable for immediate attention demands; optimal for real-time applications requiring rapid response without memory overhead.
LLaVA—Memory-Enhanced Processing	0.121	Demonstrates cognitively plausible capacity limitations under unlimited context accumulation; maintains linguistic fluency despite attention degradation.	Illustrates importance of bounded memory for maintaining cognitive alignment; useful for understanding memory interference patterns.
Gemma—Immediate Processing	0.088	Efficient processing under minimal computational demands; appropriate attention allocation for simple visual scenarios.	Suitable for resource-constrained applications; requires careful scene complexity management.
Llama—Immediate Processing	0.075	Consistent performance under stable visual conditions; sensitive to dynamic visual changes and camera movement.	Requires environmental stability for reliable performance; best suited for static interaction scenarios.
DeepSeek—Memory Integration	0.057	Sophisticated temporal context utilisation through lexical anchoring; demonstrates human-like working memory benefits.	Optimal for applications requiring temporal consistency; provides cognitive benefits in dynamic environments.
Llama—Memory Integration	0.057	Mixed temporal processing capabilities; beneficial under static conditions but degraded performance during visual dynamics.	Context-dependent reliability; requires adaptive deployment strategies based on environmental conditions.
Qwen—Immediate Processing	0.046	Functional attention allocation with multilingual cognitive flexibility; demonstrates cross-lingual processing capabilities.	Requires multilingual evaluation frameworks; potential for diverse linguistic deployment contexts.
DeepSeek—Immediate Processing	0.038	Foundation-level attention capabilities that benefit significantly from temporal context integration.	Demonstrates importance of memory augmentation; illustrates architectural potential under appropriate context conditions.
Gemma—Memory Integration	0.013	Capacity limitations prevent effective temporal context utilisation; demonstrates cognitive overload under extended context.	Highlights importance of computational capacity for memory integration; requires careful resource management.
Qwen—Memory Integration	0.000	Cross-lingual processing capabilities masked by monolingual evaluation constraints; potential cognitive alignment not captured.	Requires specialised evaluation frameworks; represents broader challenges in multilingual AI assessment.

## Data Availability

The data presented in this study are available on request from the corresponding author due to (specify the reason for the restriction).
